# Dr. Ravindra Kolhe and Dr. Smita Kolhe: The Pioneers of Improving Tribal Healthcare in Maharashtra, India

**DOI:** 10.7759/cureus.70433

**Published:** 2024-09-29

**Authors:** Amrit Mishra, Umesh Kawalkar, Aditi A Rathod, Poovvizhi Muniyasamy, Amar Mankar

**Affiliations:** 1 Community Medicine, Government Medical College Akola, Akola, IND; 2 Microbiology, Government Medical College Akola, Akola, IND; 3 Community Medicine, Datta Meghe Institute of Higher Education and Research, Wardha, IND

**Keywords:** agriculture, child health, community health services, malnutrition, poverty, social medicine

## Abstract

Dr. Ravindra Kolhe and Dr. Smita Kolhe are the pioneers of improving tribal healthcare in Maharashtra, dedicating more than three and a half decades of their lives for the sake of the people in the remote Bairagarh village of the Melghat area. Their compassionate and resilient approach and a deep sense of social justice led to a dramatic improvement in maternal and child health with a noteworthy decline in the infant mortality rate. Apart from providing affordable healthcare services, the Kolhes have been pivotal in promoting sustainable agriculture to empower the local community to improve their livelihoods. Their relentless efforts have not only enhanced healthcare facilities in Bairagarh but also addressed broader social factors such as poverty, malnutrition, and infrastructure development. In recognition of their selfless service, the couple was awarded with the prestigious Padma Shri, the fourth-highest civilian award. This narrative review article highlights their profound impact on tribal healthcare in one of Maharashtra’s most underdeveloped regions.

## Introduction and background

**Figure 1:**
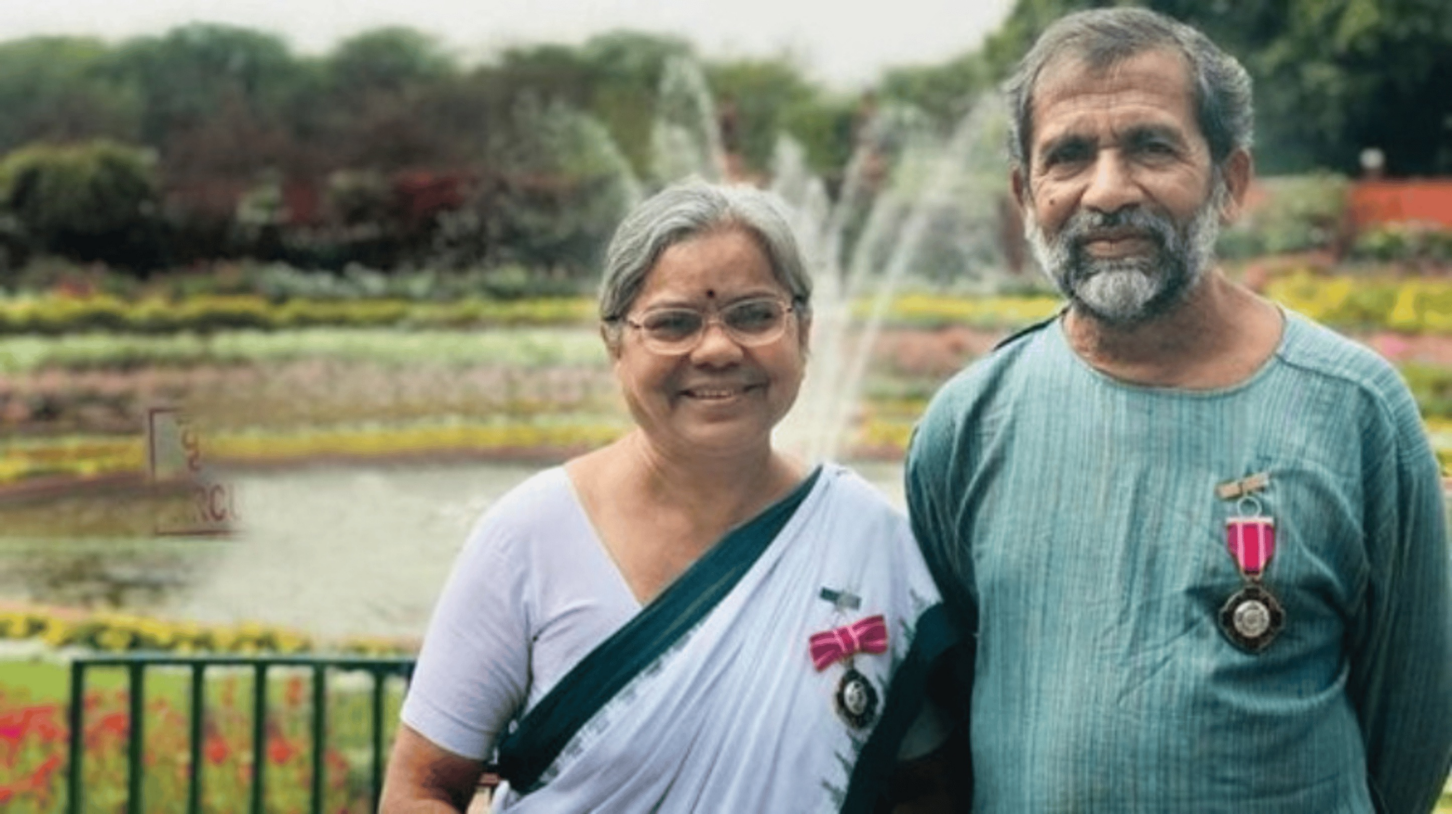
Dr. Ravindra Kolhe and Dr. Smita Kolhe awarded with the Padma Shri award. Image courtesy: Permission obtained from Dr. Ravindra Kolhe and Dr. Smita Kolhe.

Dr. Ravindra Kolhe and Dr. Smita Kolhe (Figure 1) are a dedicated husband-and-wife team of doctors, who have become icons for the transformation of tribal health in Maharashtra, India. For more than three and a half decades, the couple has served the tribal communities of the Bairagarh region in Melghat, Maharashtra. It was an area known not only for its challenging terrain but also for extreme poverty, high rates of malnutrition, and infant mortality [1]. Their work has provided essential and affordable medical services along with sustainable and community-based agriculture for the tribal population [2]. In 2019, the Government of India recognized their work and awarded them with the fourth-highest civilian award, the Padma Shri [3].

## Review

Early life and education

Dr. Ravindra Kolhe was born in 1960. In his early years, he was inspired by the works of Mahatma Gandhi and Vinoba Bhave. He completed his MBBS degree from Government Medical College and Hospital, Nagpur, Maharashtra, in 1982. One of his college professors, Dr. Jaju, instilled the pre-requisite mantra for practicing in remote areas, stating that to work as a doctor in remote areas, one must learn the three essential medical skills, namely conducting labor and delivery without the facilities such as ultrasonography and blood transfusion, making a diagnosis of pneumonia without X-Ray facility, and, lastly, mastering the cure for diarrhea in resource-limited settings. He was driven by a strong will to deliver social justice and that led him to use his medical knowledge to serve marginalized communities [4].

The call to Melghat

**Figure 2:**
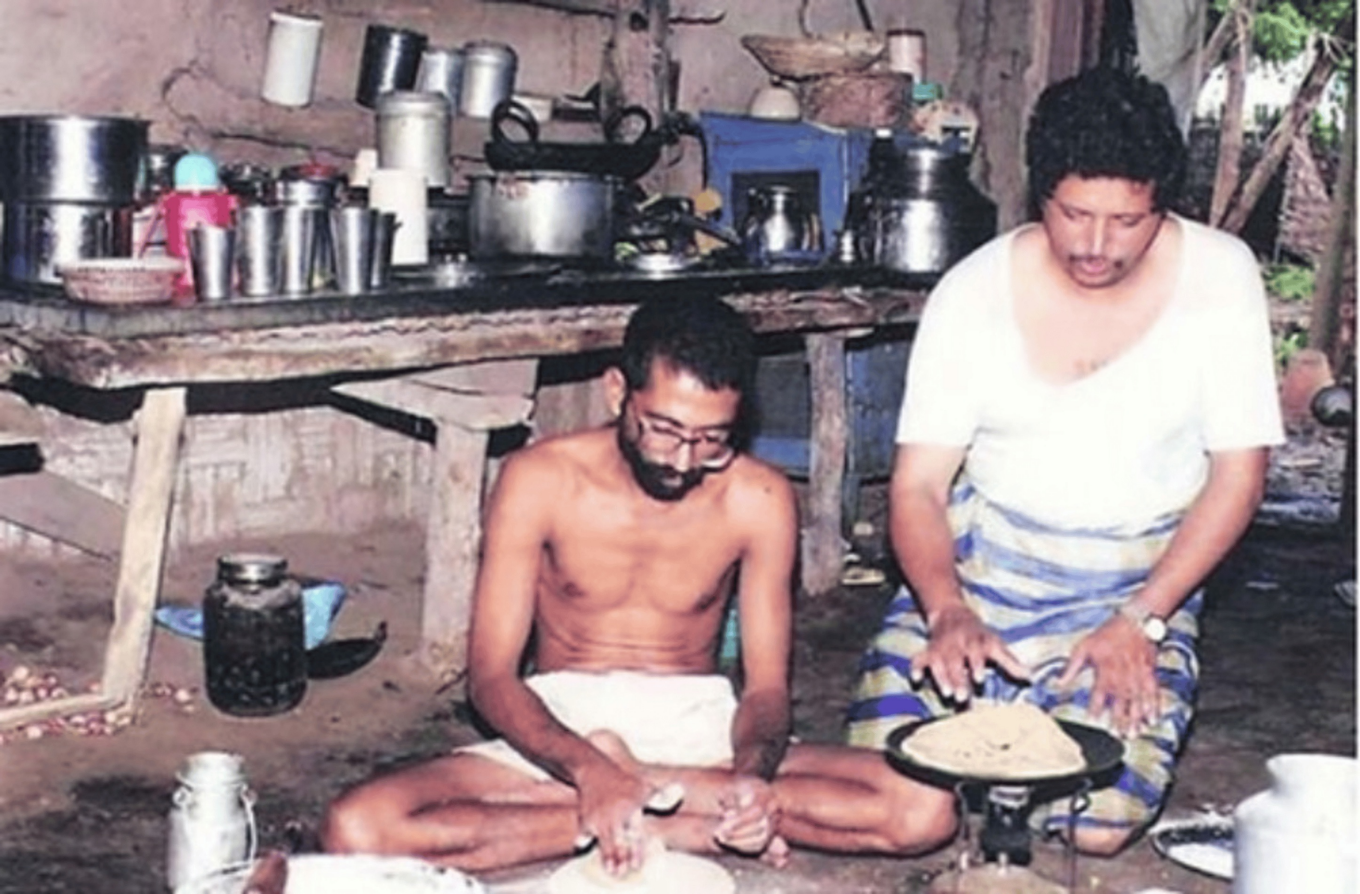
The one-rupee doctor living in his house at Bairagarh Image courtesy: Permission obtained from Dr. Ravindra Kolhe and Dr. Smita Kolhe.

After Dr. Kolhe graduated, he worked as a House Officer during 1984 to 1985. He then decided to explore the remote areas of Maharashtra and came across Bairagarh, a part of Amravati district. Bairagarh presented itself with significant challenges, such as geographical isolation, as the roadways of Amravati district ended at Harisal, and, thereafter, a person wishing to travel to Bairagarh had to travel 40 kilometers by foot. He started his medical practice at Bairagarh in 1985 for a meagre initial consultation fee of Rs. 2 and subsequent follow-up at Rs. 1 only. He noted the extreme poverty and lack of basic infrastructure in the region [2]. He lived in a simple house with basic amenities (Figure 2) [5].

**Figure 3:**
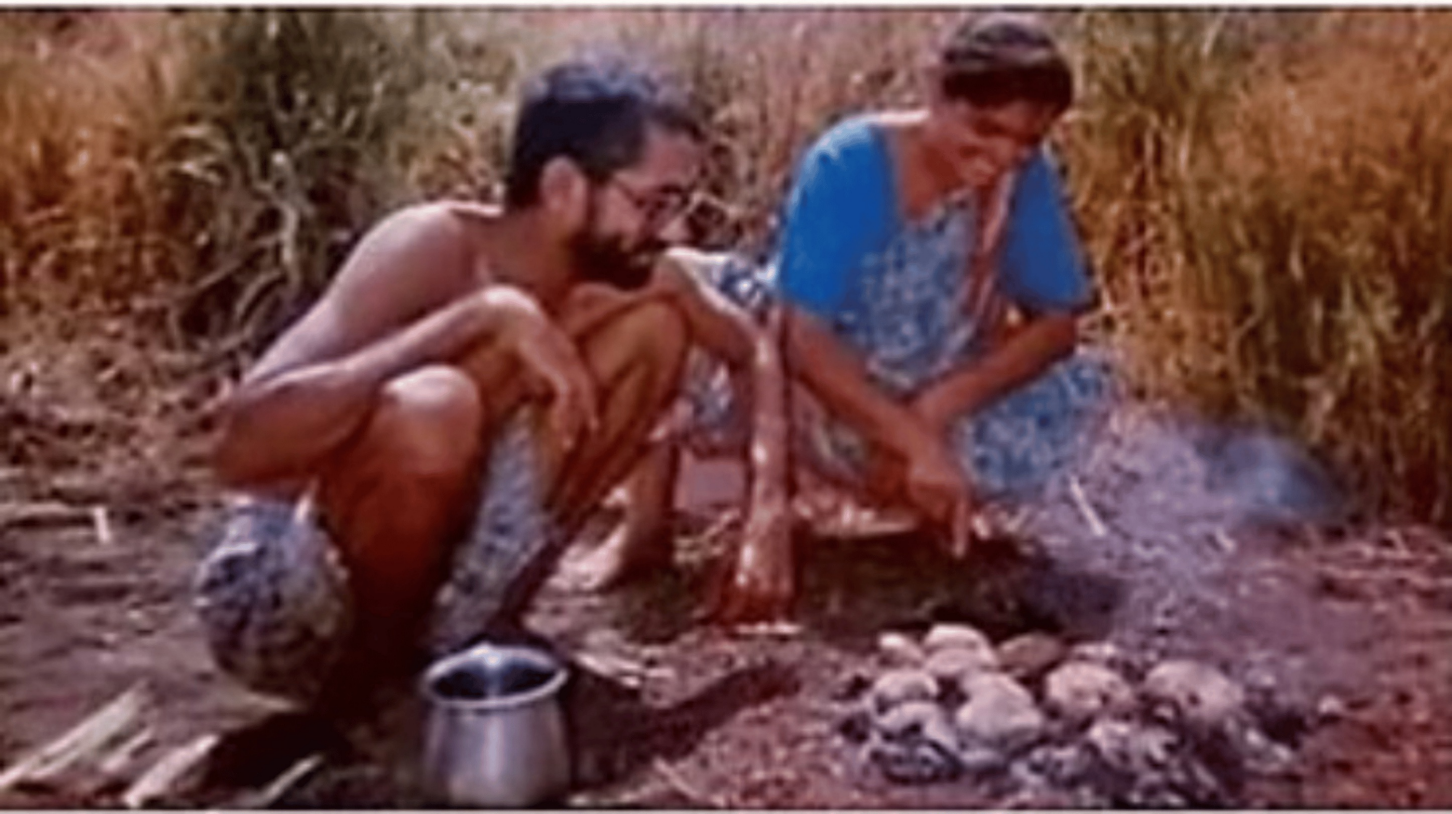
The Kolhe couple in their early days at Bairagarh Image courtesy: Permission obtained from Dr. Ravindra Kolhe and Dr. Smita Kolhe.

An incident during his second week of medical practice turned his focus on seeking postgraduate expertise. He was faced with a crisis of a tribal man losing his hand in a blast; he realized that it was difficult as a young practitioner to face this situation. He realized that effective healthcare delivery in Bairagarh required him to pursue higher studies. He returned to Nagpur with a vow to complete an MD postgraduation degree before restarting his service at Bairagarh. In 1988, he completed the MD Degree in Preventive and Social Medicine and decided to get married before venturing back to Bairagarh. He had several conditions for a suitable partner, namely an understanding partner willing to face the hardship of village life, trekking 40 kilometers on foot, being comfortable with a non-lavish court marriage at Rs 5, being headstrong to run a household with Rs. 400 (his monthly income from the medical practice), and should be unashamed to beg for the welfare of others. He found his life partner in Dr. Smita Manjare (Figure 3), a homeopathic practitioner at Nagpur. She had a degree in law and a certification in yoga therapy. Together, the couple ventured to Bairagarh with a deep desire to improve the health outcomes of the tribal community [4]. The Kolhe couple lived without much basic amenities in their early days at Bairagarh [5].

**Figure 4:**
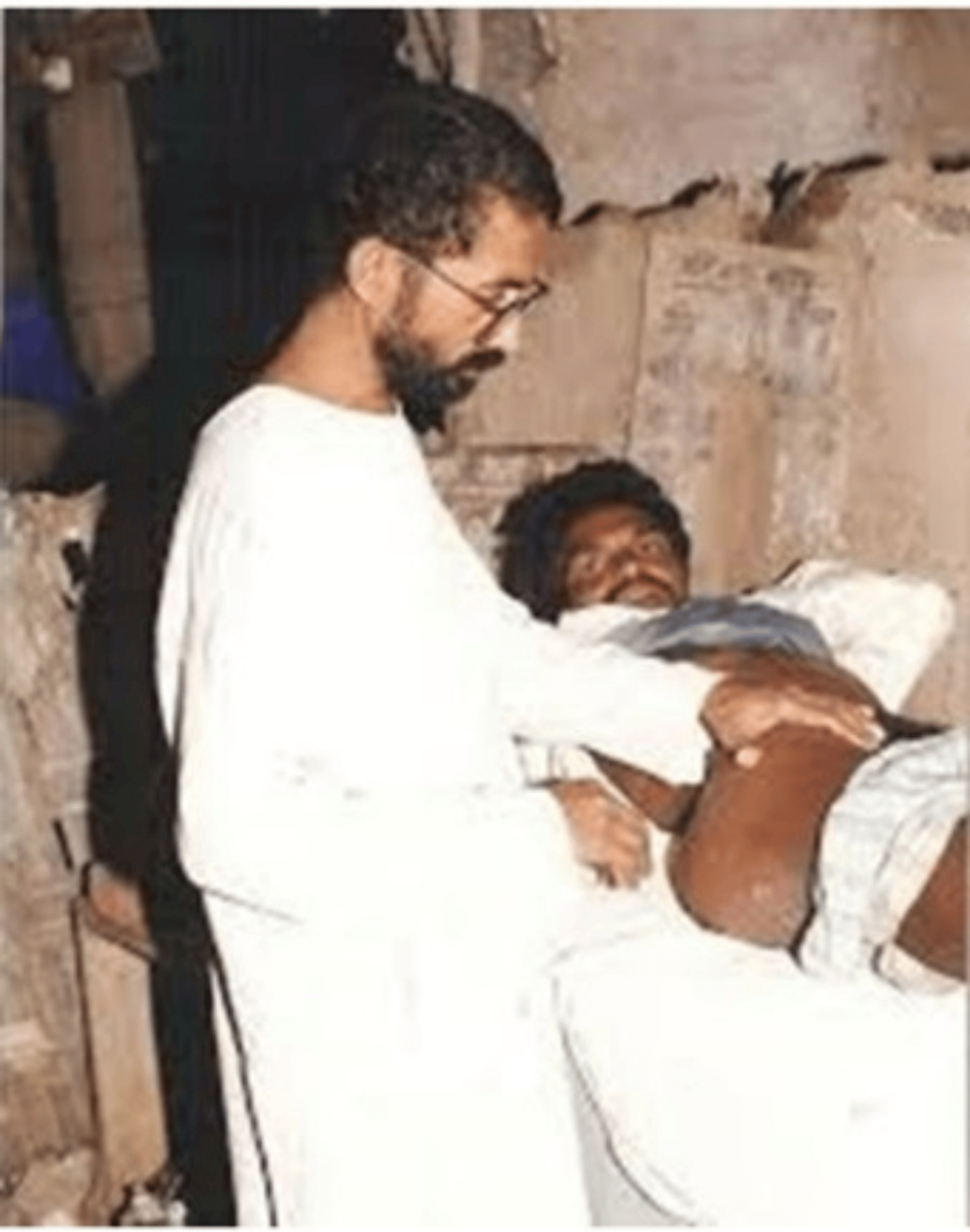
Dr. Ravindra Kolhe examining a patient in the village. Image courtesy: Permission obtained from Dr. Ravindra Kolhe and Dr. Smita Kolhe.

Initially, the people of Bairagarh were hesitant to Dr. Smita as she was a firm fighter for women’s empowerment. However, after two years, the couple expected their first child, but, unfortunately, they faced postnatal complications, with the infant being affected with severe pneumonia, meningitis, and septicemia. They were presented with the option to refer their child to the district hospital of Akola, but Dr. Smita decided to continue treating their child with the limited resources available at Bairagarh, winning the respect and the approval of the villagers [4]. The one-rupee doctor was a savior of the people of Bairagarh (Figure 4) [5].

Impact on infant mortality rate

The Kolhes aimed their focus on improving the maternal and child health of the tribal people. In 1990, the infant mortality rate (IMR) was a whopping 200 deaths per 1,000 live births. The reasons for mortality were many, including but not limited to poverty and malnutrition. Through interventions such as improved prenatal and postnatal care, they were successful in reducing the IMR to less than 40 deaths per 1,000 live births over time [2].

Empowerment of local communities

The Kolhes’ work on the empowerment of tribal communities has not only been through medical service but also through sustainable agricultural practices. With the improvement in their health, the villagers asked the couple to assist them with the issues of cattle and farming. To acknowledge this unusual request, Dr. Ravindra Kolhe had to resort to studying agriculture at the Dr. Panjabrao Deshmukh Krishi Vidyapeeth (PDKV) Institute, Akola, and also about veterinary science from his friend [2].

He developed fungus-resistant seeds through his research; however, none of the villagers were volunteering to try cultivating them. Thus, the Kolhes (with their elder son) started farming by themselves in 2005, with the main focus on soya plantation. They initiated a profit-oriented farming for the villagers and brought about contemporary cultivation practices for cash crops such as ginger, garlic, turmeric, and watermelon. They progressed to forest conservation and helped farmers predict upcoming droughts [2].

**Figure 5:**
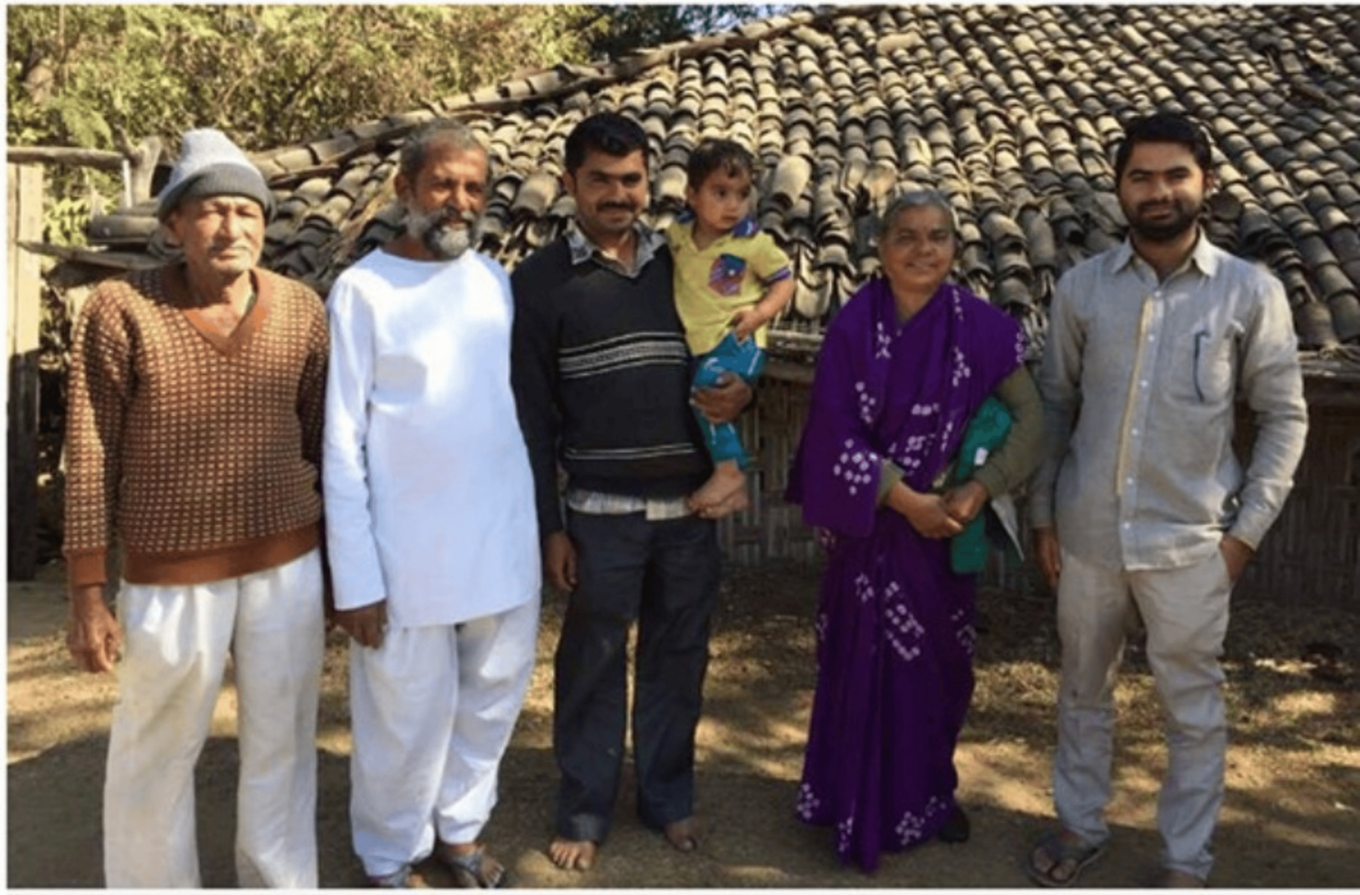
The Kolhe family. From left: Shri Devrao Kolhe (Dr. Ravindra Kolhe’s father), Dr. Ravindra Kolhe, Dr. Rohit (elder son), Master Viraj (grandson), Dr. Smita Kolhe and Dr. Ram (younger son). Image courtesy: Permission obtained from Dr. Ravindra Kolhe and Dr. Smita Kolhe.

The family (Figure 5) also took up the task of improving the Public Distribution System (PDS) to ensure adequate rations to the villagers in times of crisis. The Minister of Public Works Department visited them after hearing about their social service and wished to build a house for them, but it was Dr. Smita who insisted that the village must have access to good roads, electricity, and infrastructure. This paved the way for improvement of infrastructure and making Bairagarh as farmer-friendly [4].

The Kolhes organize “Karunai” camp twice yearly, focusing on the youths, to raise awareness about novel environmental-friendly farming techniques, valuable government schemes for farmers, and information about medical services including HIV/AIDS, to name a few. Thousands of volunteers attend it in both online and offline modes [2]. The Kolhes still fervently strive to bring about better facilities and uninterrupted supply of electricity to all the villages in the Melghat region. The legacy of the one-rupee doctor will continue to inspire healthcare professionals worldwide and emphasize the importance of addressing disproportionate healthcare in tribal areas.

## Conclusions

Through their compassionate approach, Dr. Ravindra and Dr. Smita Kolhe have not only improved the healthcare of the people of Bairagarh but also demonstrated sustainable agricultural solutions to empower them. Their inspiring impact is a powerful example of what can be accomplished when healthcare is rooted in community engagement and cultural understanding. The people who lived in poverty, without basic amenities such as electricity and proper roads for many years, currently have access to electricity, better roads and transportation, and dedicated health centers.
